# Inner ear hair cells deteriorate in mice engineered to have no or diminished innervation

**DOI:** 10.3389/fnagi.2015.00033

**Published:** 2015-03-18

**Authors:** Jennifer Kersigo, Bernd Fritzsch

**Affiliations:** Department of Biology, University of IowaIowa City, IA, USA

**Keywords:** inner ear, hair cells, degeneration, innervation, neurotrophins, conditional deletion

## Abstract

The innervation of the inner ear critically depends on the two neurotrophins Ntf3 and Bdnf. In contrast to this molecularly well-established dependency, evidence regarding the need of innervation for long-term maintenance of inner ear hair cells is inconclusive, due to experimental variability. Mutant mice that lack both neurotrophins could shed light on the long-term consequences of innervation loss on hair cells without introducing experimental variability, but do not survive after birth. Mutant mice with conditional deletion of both neurotrophins lose almost all innervation by postnatal day 10 and show an initially normal development of hair cells by this stage. No innervation remains after 3 weeks and complete loss of all innervation results in near complete loss of outer and many inner hair cells of the organ of Corti within 4 months. Mutants that retain one allele of either neurotrophin have only partial loss of innervation of the organ of Corti and show a longer viability of cochlear hair cells with more profound loss of inner hair cells. By 10 months, hair cells disappear with a base to apex progression, proportional to the residual density of innervation and similar to carboplatin ototoxicity. Similar to reports of hair cell loss after aminoglycoside treatment, blobbing of stereocilia of apparently dying hair cells protrude into the cochlear duct. Denervation of vestibular sensory epithelia for several months also resulted in variable results, ranging from unusual hair cells resembling the aberrations found in the organ of Corti, to near normal hair cells in the canal cristae. Fusion and/or resorption of stereocilia and loss of hair cells follows a pattern reminiscent of Myo6 and Cdc42 null mice. Our data support a role of innervation for long-term maintenance but with a remarkable local variation that needs to be taken into account when attempting regeneration of the organ of Corti.

## Introduction

It is estimated that over 900 million people worldwide will have at least a 25 dB reduction in hearing sensitivity by 2025 [http://hearinghealthmatters.org/hearinginternational/2011/incidence-of-hearing-loss-around-the-world/]. Even mild hearing loss (26–40 dB HL) may deprive people from their accustomed way of communication (Yamasoba et al., 2013), promote cognitive decline (Lin et al., 2013), and possibly increase the risk for developing dementia, including Alzheimer's disease (Lin and Albert, 2014). Clinically, hearing loss is multifactorial in its etiology, having both genetic and environmental (noise exposure, ototoxic drugs, neurotoxic drugs, etc.) components (Kopecky and Fritzsch, 2011; Makary et al., 2011; Huisman and Rivolta, 2012; Rivolta, 2013). Sensorineural hearing loss is a common type of age-related hearing loss (AHL) and mainly results from loss of cochlear hair cells (HCs) and/or spiral ganglion neurons (SGNs). Potentially more devastating is a decline in vestibular function which typically starts about a decade after the onset of hearing loss (Rauch, 2001). Vestibular dysfunction can increase the risk of falling, resulting in fractures and subsequent morbidity of the elderly. Recently the hallmarks of aging were reviewed and cell communication was found to be a major aspect that defines which cell will survive and which will die (López-Otín et al., 2013), possibly underlying the diversity of cellular reactions that has been stressed in recent papers studying hair cell loss after ototoxic treatments (Taylor et al., 2012). Here we evaluate the historically controversial influence of innervation on hair cell viability in the ear using newly developed models of targeted deletion of neurotrophins.

Human temporal bone studies of the cochlea suggest that neuronal and HC loss are unrelated as over 90% of neuronal loss can occur without loss of HCs (Otte et al., 1978; Makary et al., 2011). Animal studies also suggest that innervation and HC loss can happen independently of each other (Perez and Bao, 2011; Kidd and Bao, 2012) except for inconclusive data that report the loss of some HCs after afferent innervation to the inner ear was cut (Sugawara et al., 2005). In the vestibular system of humans, there appears to be a somewhat matching decline of both vestibular ganglion neurons (VGNs) and HCs over time (Rauch, 2001). Earlier claims in studies involving adult guinea pigs show complete loss of HCs in vestibular sensory epithelium 4 months after vestibular nerve transection (Favre and Sans, 1991) have not been confirmed in other investigations in humans (Suzukawa et al., 2005) leaving the loss of vestibular hair cells after loss of innervation open to interpretation. To date, a large portion of the studies addressing the dependency of inner ear hair cell survival on innvervation utilize surgical techniques with potential flaws: either incomplete surgical denervation or inadvertent disruption of blood supplies may affect data (Sugawara et al., 2005). Despite over 50 years of work on this subject, it is fair to say that no unequivocal answer has been reached largely due to technical limitations in all but one study that shows complete loss of all vestibular hair cells after surgical denervation (Favre and Sans, 1991).

Of note, in contrast to these disputed effects related to surgical removal of innervation on adult hair cells, data on mutant mice that lack all innervation to the ear by various mutations have established that absence of innervation has no short-term effect on HC development (Fritzsch et al., 1997a; Ma et al., 2000; Yang et al., 2011). In fact, removing neurotrophin receptors eliminates innervation without affecting hair cell development (Fritzsch et al., 1997a). This absence of any apparent effect of denervation contrasts with all other sensory cells, which seemingly depend on innervation either for complete differentiation or viability (Fritzsch et al., 1998). For example, severing gustatory nerves results in rapid loss of taste bud sensory cells, which can reappear after nerve fibers grow back into the skin (Farbman, 2003; Fei et al., 2014). However, some embryonic differentiation of taste sensory cells can occur in the absence of innervation (Fritzsch et al., 1997b; Ito et al., 2010) and gustatory nerve fibers cannot induce taste buds if the molecular competence of the epidermis is changed by mutating *Sox2* (Okubo et al., 2006, 2009). These data suggest that initial formation of taste sensory cells occurs autonomously, much like hair cells in the ear (Ma et al., 2000) but innervation is needed to maintain sensory cells. Similar to taste buds, electroreceptive sensory cells and organs depend on innervation for maintenance. Hair cells die within hours after severing the nerve and organs disappear rapidly through cell death after nerve fibers are cut and reappear rapidly upon reinnervation (Fritzsch et al., 1990). Transplantation studies have shown that the initial development of electroreceptive organs may be autonomous (Northcutt et al., 1995), again suggesting that afferents maintain but do not induce electroreceptive sensory cells. Neither taste buds nor electroreceptors have an efferent innervation, clearly indicating the role of afferents for maintenance. Electroreceptive sensory cells are closely related to the mechanosensory HCs of the lateral line, neuromasts (Duncan and Fritzsch, 2012), which also seem to depend on innervation for long term viability. In bony fish and amphibians, the hair cells of neuromasts are lost after months of denervation (Jones and Singer, 1969). These data suggest that possibly all placode derived sensory cells can differentiate autonomously but require innervation for viability. Among placode derived sensory cells, inner ear hair cells appear to be unique: like other placode derived sensory cells they have autonomous development in the absence of innervation but may not depend on afferent innervation for long term viability.

A new approach using a transgenic mutation resulting in the targeted deletion of neurotrophins to test the potential influence of afferents and efferents on HC viability, without compromising blood supply, could clarify this issue. Previous work has shown that mice lacking neurotrophins or their receptors are born with little to no innervation (Ernfors et al., 1995; Silos-Santiago et al., 1997; Fritzsch et al., 2004) but these mice die soon after birth. Mice with ear-specific conditional deletions of the two relevant neurotrophins in the ear (*Pax2-cre; Ntf3^f/f^*, *Bdnf^f/f^*) are viable for several months but are deaf and show vestibular and cerebellar motor control defects. We raised these mice for up to 10 months and investigated the pattern of remaining HCs and innervation using Myo7a, tubulin, and neurofilament (NF) immunohistochemistry, myelinated nerve fiber staining, and SEM. These mice show an age-dependent, progressive loss of HCs that correlates with the reduction of innervation in the cochlea and vestibular organs and suggests a yet to be determined, variable threshold of innervation for different organs and different hair cells within a given organ.

## Material and methods

### Mouse breeding and collection

*Pax2-cre* mice (Ohyama and Groves, 2004) were crossed with floxed *Ntf3* (Bates et al., 1999) (aka *NT3*) and floxed *Bdnf* mice (Gorski et al., 2003) to generate conditional, ear-specific and viable mutants that lack neurotrophin expression in the ear. Breeding pairs consist of mice carrying the *Pax2-cre* together with heterozygosity of the floxed neurotrophins (*Pax2-cre; Ntf3^f/+^; Bdnf^f/+^*). These mice were crossed with mice homozygotic for floxed alleles of both neurotrophins (*Ntf3^f/f^; Bdnf^f/f^*). 1 in 8 mice were doubly homozygotic for both floxed genes and also expressed cre. Combinations of cre with heterozygotic floxed *Bdnf* and homozygosity of *Ntf3* (*Pax2-cre; Ntf3^f/f^; Bdnf^f/+^*) or homozygosity for floxed Bdnf and heterozygosity for Ntf3 (*Pax2-cre; Ntf3^f/+^; Bdnf^f/f^*) are also included here. These mice lose much of their innervation of the organ of Corti and in case of loss of all *Bdnf*, also all innervation to canal cristae (Fritzsch et al., 2004). Because of the further delay in innervation loss in the apical half of the cochlea, we concentrated on the basal turn for this presentation except where stated differently.

Mice were genotyped within 3 days after birth. Non-desired littermates were eliminated to increase the viability of vestibular defected mutant mice (due to loss of Bdnf). Mice were raised to the designated age of 1, 2, 4, 7–10 months and were sacrificed. Six mutant animals were collected per the three genotypes whenever possible together with age-matched control littermates at the designated age to minimize genetic background effects. Data were pooled across two or more litters to eliminate any genetic background bias. Given that we had to cross three distinct mutant lines carrying the Pax2-cre, the floxed Bdnf and the floxed Ntf3 into a mixed mouse line, we do not expect strain specific effects of time delay as previously reported (Taylor et al., 2012). We cannot exclude that some strain specific background effects are present in our mixed lines. Nevertheless, we consider the best comparison to be with littermates with a different genotype but housed under identical circumstances in the same box to avoid undo bias introduced by unknown genetic background effects.

For the present analysis we concentrated on three genotypes: *Pax2-cre; Ntf3^f/f^; Bdnf^f/f^; Pax2-cre; Ntf3^f/f^; Bdnf^f/+^*and *Pax2-cre; Ntf3^f/+^; Bdnf^f/f+^*. The latter two genotypes had each only one single allele of neurotrophin left whereas the first had no neurotrophin expression left in the ear. In every case we used age matched controls to compare the effect of mutations. Animals without a cre or without floxed neurotrophin alleles were designated as control animals.

Control and mutant mice were raised together to a defined age, euthanized by deep anesthesia with an intraperitoneal injection of Avertin (1.25% tribromoethanol solution; 0.025 ml/g of body weight). Absence of blink and paw withdrawal reflex was used as evidence for proper depth of anesthesia. Once all reflexes had seized the chest was opened and the mouse was transcardially perfused with 4% paraformaldehyde (PFA) in 0.1 M Phosphate buffer using appropriate-sized needles assuring immediate death. After perfusion, ears were dissected and fixed overnight in 4% PFA and decalcified in 10% EDTA for 2–3 weeks, depending on age. The method of anesthesia is consistent with *AVMA Guidelines on Euthanasia* and is approved by the University of Iowa Institutional Animal Care and Use Committee (IACUC; protocol #1403046).

### Immunostaining

Cochleae were micro-dissected, split into near equal sized basal and apical turn sections and immunostained for Myo7a (HCs), tubulin and neurofilament (neuronal processes). Cell nuclei were stained with Hoechst stain according to existing protocols (Jahan et al., 2013).

### Lipophilic dye tracing

Small pieces of dye-soaked filter paper (Fritzsch et al., 2005a; Tonniges et al., 2010) were implanted into the cochlear nuclei (NeuroVue™ Maroon) or the efferent fiber tract (NeuroVue™ Red) in animals fixed in 4% PFA. Fixed tissue was kept at 60°C for ~48–96 h to allow for dye diffusion from the hindbrain to the ear. Ears were micro-dissected, split into a basal and apical turn and mounted on a slide in glycerol. To avoid diffusion of dye out of the lipid bilayer into the glycerol used as mounting medium, images were taken immediately with a Leica TCS SP5 confocal microscope.

### Imaging

Immunostained cochlea halves were mounted flat on a slide using glycerol, coverslipped and imaged using a Leica SP5 confocal microscope. Data sets were generated by collecting stacks at 3–6 μm steps (depending on the magnification) in 100–200 μm long segments at three different positions: the basal hook region, near the apical tip and at approximately the middle of the cochlea.

### SEM imaging

Selected ears of animals at late stages of HC loss were imaged using SEM to detail the loss and aberration of hair bundles and the reorganization of supporting cells after induced HC loss as recently described (Jahan et al., 2010). Ears designated for SEM were postfixed in 2.5% glutaraldehyde followed by 1.0% OsO4 fixation. The cochlea apex was cut away from the cochlear base with fine scissors resulting in an apical turn and a basal ¾ turn. OsO4 stains all myelinated nerve fibers black and images were taken after OsO4 staining to verify completion of nerve fiber loss or partial loss, depending on the genotype. Images of osmicated ears were taken on a Leica dissection scope using identical settings for mutant and control animals. SEM preparations were critical point dried, sputter coated and viewed with a Hitachi S-4800 scanning electron microscope. HC and supporting cell reorganization were interpreted according to known effects of aminoglycoside toxicity (Taylor et al., 2012). Cochlea and vestibular organs of age matched control and mutant littermates were processed together but differed in being either left or right ear for easy identification.

### *In situ* hybridization

Both the floxed *Bdnf* and the floxed *Ntf3* have been used in the ear for targeted deletion but only the Bdnf has been used before with Pax2-cre (Zilberstein et al., 2012; Zuccotti et al., 2012). We therefore verified the absence of *Ntf3* in Pax2-cre mice at birth to show that indeed there was no detectable level of Ntf3 at this late stage, consistent with the innervation phenotype. *In situ* hybridization was performed as described previously (Duncan et al., 2011) using a probe specific for *Ntf3* (courtesy of L. Reichardt). Previous work has already demonstrated the effectiveness of Pax2-cre to excise the floxed alleles of *Bdnf* (Zuccotti et al., 2012) and the effects agreed with previously described losses of *Bdnf*.

## Results

### Complete absence of the neurotrophins *Ntf3* and *Bdnf* (*Pax2-cre; Ntf3^f/f;^ Bdnf^f/f^)*

**Figure 1:**
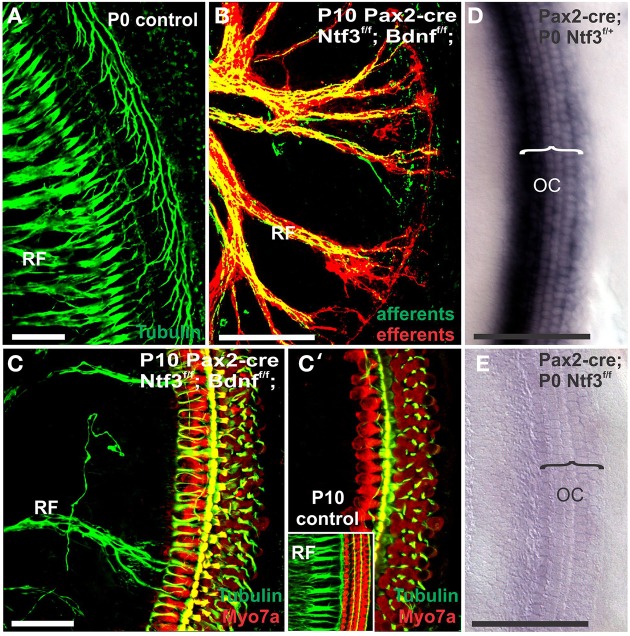
**Conditional deletion of floxed *Ntf3* and floxed *Bdnf* in the ear using *Pax2-cre* leads to near complete loss of afferents by P10 (B,C,C′) compared to innervation in a control at birth (A) or 10 days (insert in C′) indicating it is a true loss of afferents and not just delayed innervation**. Lipophilic dye labeling reveals that efferent fibers (red) reach to the IHCs **(B)** but also form tunnel-crossing fibers to OHCs. Afferents (green in **B**) are nearly absent and reduced to few radial bundles in the middle turn. Immunostaining for tubulin (green in **B**) shows very few fibers (both afferents and efferents) left in the middle turn **(C)** but not in the base **(C′)**. Note that all hair cells, immunostained for Myo7a, are normally developed and surrounded by supporting cells (stained in green with anti-tubulin). *In situ* hybridization for *Ntf3* **(D,E)** shows a strong signal in control animals **(D)** but no signal above background after conditional deletion of Ntf3 using Pax2-cre **(E)**. Bar = 100 μm **(A,B,D,E)** and 50 μm in **(C,C′)**.

**Figure 2:**
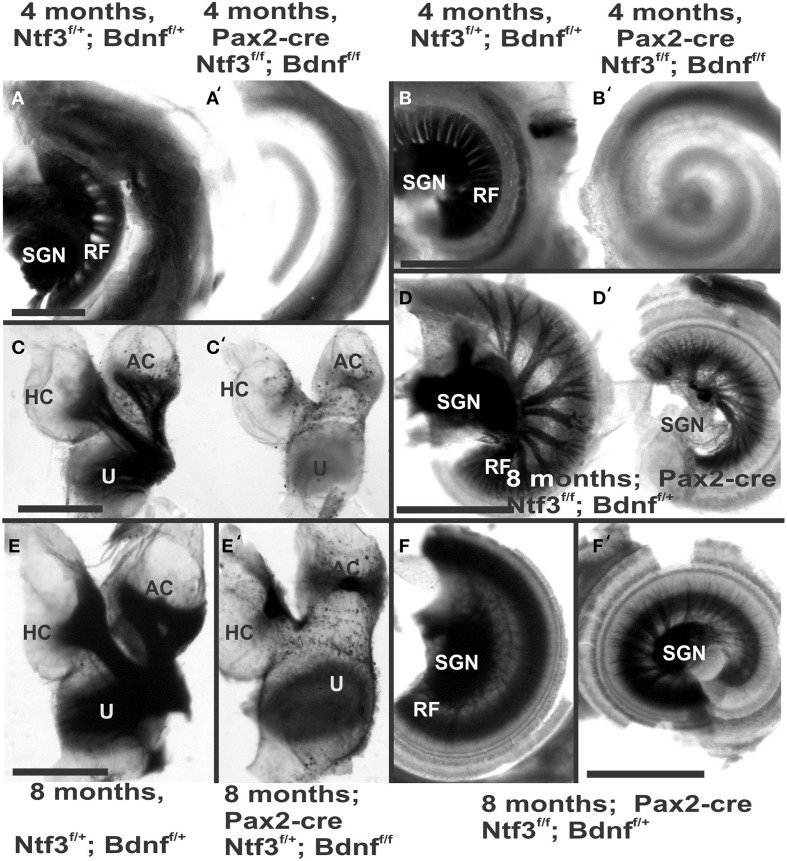
**Osmication labels all myelinated nerve fibers and is used here to assess completeness of nerve fiber loss**. Inner ears of the 4 month old control **(A,B,C)** and *Pax2-cre; Ntf3^f/f;^ Bdnf^f/f^* mutant mice **(A′,B′,C′,D,D′)** of 7 months old *Pax2-cre; Ntf3^f/f;^ Bdnf^f/+^* mutant mice, and **(E,F)** are inner ears of 7 months old control and *Pax2-cre; Ntf3^f/+;^ Bdnf^f/f^* mutant mice. Note the myelin in the spiral ganglion neurons (SGN) in the control littermate **(A,B)** and complete absence of any myelin staining in both the basal **(A′)** and apical turn **(B′)** of mice with a conditional deletion of both neurotrophins **(A′,B′)**. Likewise, there are no myelinated nerve fibers to the horizontal (HC) or anterior canal cristae (AC) of the conditional mutants **(C′)** in stark contrast to the control littermate **(C)**. *Pax2-cre; Ntf3^f/f;^ Bdnf^f/+^* has some innervation remaining after 11 weeks to the middle turn **(D)** and apex **(D′)** while the basal hook region (top left in **D**) is devoid of any radial fibers. *Pax2-cre; Ntf3^f/+;^ Bdnf^f/f^* mutants have no nerve fibers to the canal cristae **(E,E′)** and reduced innervation to the base and the apex **(F,F′)**. These data show that a single allele of *Bdnf* provides enough support to rescue many neurons for at least several weeks **(D,D′)** and that a single allele of Ntf3 is less effective for long term innervation (compare **D,F**). Bar indicates 500 μm.

Mice without any neurotrophins were difficult to maintain past postnatal day 21 (P21). Morbidity past 2 months was very high causing loss of all but one animal collected at 4 month of age. Morbidity may relate to aberrations in the cerebellum previously demonstrated in mutants lacking both neurotrophin receptors (Silos-Santiago et al., 1997). Given the presence of the neurotrophin *Ntf3* in cochlear nuclei (Maricich et al., 2009) and the delayed expression of *Pax2-cre* in the cochlear nuclei (Ohyama and Groves, 2004), more afferent fibers should survive past birth compared to the neurotrophin double null mutant mice which lose all innervation at or around birth, depending on the background (Ernfors et al., 1995; Yang et al., 2011). Indeed, P10 mice had limited afferent and more profound efferent supply to the cochlea (Figure 1) labeled through selective application of lipophilic dyes to cochlear nuclei and efferent fibers bundles, respectively (Simmons et al., 2011). No afferent or efferent fibers were detected in these animals in the basal turn. Immunostaining for tubulin also showed limited innervation at P10. Development of HCs (shown by Myo7a immunostaining) appeared normal (Figure 1). Likewise, no alteration in supporting cells could be detected using the immunostaining for tubulin (Figure 1). Complete absence of any innervation in these conditional null mutants for both neurotrophins (*Pax2-cre; Bdnf^f/f^; Ntf3^f/f^*) seemed to occur around P21 as neither tracing nor immunstaining for tubulin or neurofilament showed any fiber to the ear in these (data not shown) and older mice (Figure 2). However, even at this stage there was no change in hair cells, suggesting that at least until P21 partial or complete afferent and efferent loss has no effect on early differentiation of hair cells in the organ of Corti or the vestibular organs.

The most profound effect of the complete loss of innervation was in a 4 month old double conditional null mouse, the oldest mouse of this genotype obtained thus far. Absence of innervation was verified with OsO4 as previously described (Retzius, 1884; Postigo et al., 2002). OsO4 labels all myelinated nerve fibers in control littermates including a high density of radial fiber bundles connecting the spiral ganglion neurons with the organ of Corti (Figures 1A,B). Neither the cochlea (Figures 2A′,B′) nor the vestibular region (Figure 2C) showed any myelinated nerve fibers in the mutant mice, confirming the complete absence of innervation of vestibular and auditory organs by myelinated nerve fibers. Neither neurofilament and tubulin nor Myo7a showed positive immunostaining (data not shown). However, such negative results could be due to numerous problems, including loss of hair cells and fibers or their ability to express such epitopes in detectable amounts. We next investigated the degree of differentiation of the organ of Corti HCs using SEM.

**Figure 3:**
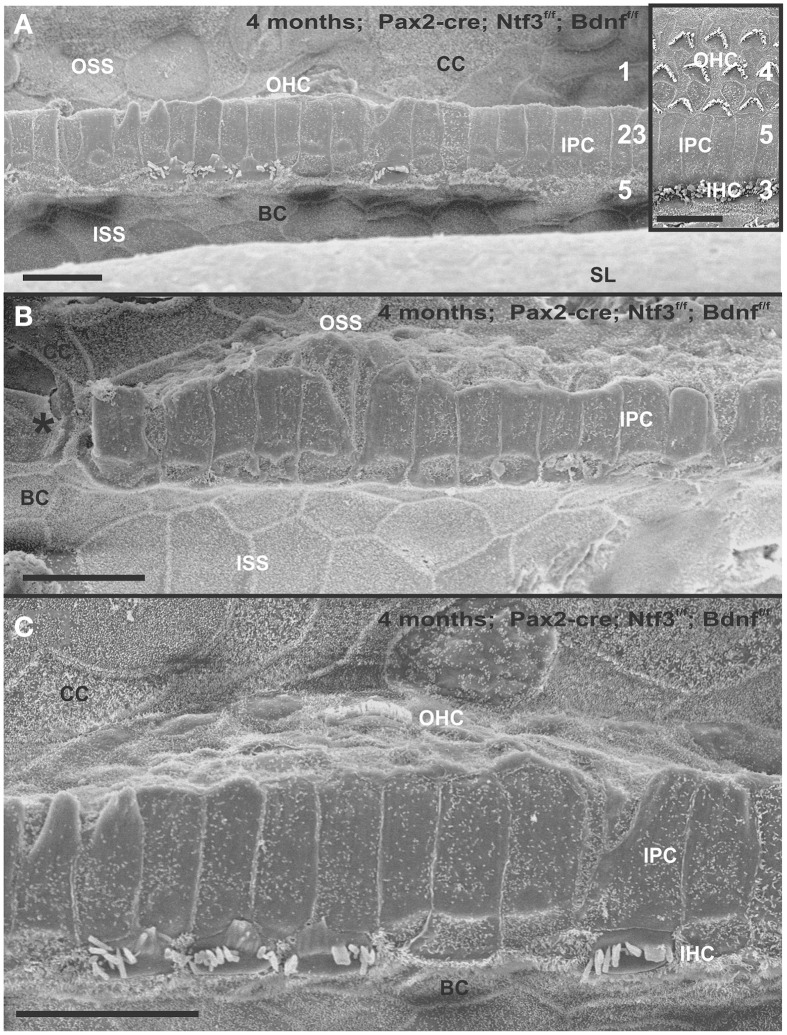
**Four month old *Pax2-cre; Ntf3^f/f^; Bdnf^f/f^* mutant shows partial or complete loss of HCs along the organ of Corti (A–C) that contrasts sharply with control littermates that have no obvious defects in HCs (insert in A)**. Except for small regions (asterisk in **B**) where border cells (BC) of the inner spiral sulcus (ISS) seem to approximate Claudius cells (CC) of the outer spiral sulcus (OSS), inner pillar cells (IPCs) are present even in areas lacking all hair cells. Outer hair cells (OHC) are mostly lost at this stage in the basal turn whereas inner hair cells (IHC) show partial loss. Numbers in **(A)** indicate remaining HCs (5) and IPCs (23) that should normally form a ratio of IPC:OHC:IHC of 5:4:3 as in control animals (insert in **A**). In mutants this ratio is 7: 3:1. **(C)** shows a tilted and enlarge version of **(A)** to reveal the single OHC barely visible on the steep slope of the reticular lamina. Bar equals 10 μm.

**Figure 4:**
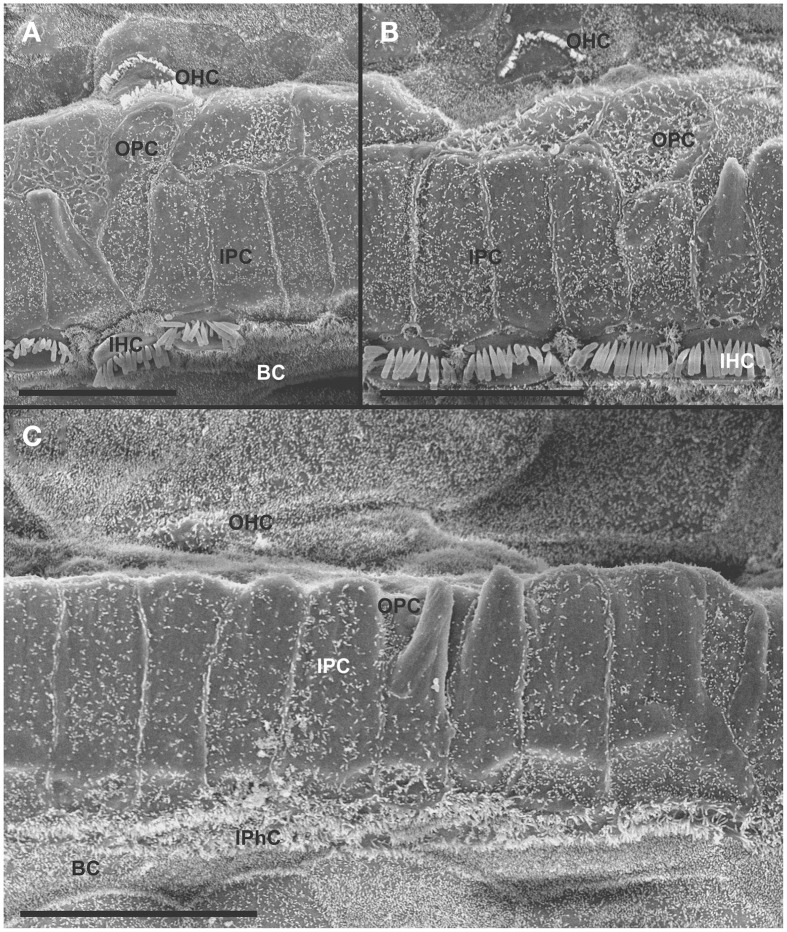
**Details of the organ of Corti reorganization after 4 months of denervation indicate an uncoupling of changes of IPCs from either IHC or OHC loss**. IPC protrusions above the underlying outer pillar cells (OPC) can be seen in the presence **(A,B)** or absence **(C)** of either IHCs, OHCs, or both IHCs and OHCs **(C)**. Note that loss of the first row of OHCs leads to an expansion of OPCs that becomes continuous **(A,B)**. In certain areas, cells are present between IPCs and BCs **(C)** with dense, short microvilli resembling the inner phalangeal cells (IPhC) between IHCs **(B)**. In other areas, medial expansions of the reticular head of IPCs seem to be in direct contact with border cells (BC in **A**). Bar equals 10 μm.

**Figure 5:**
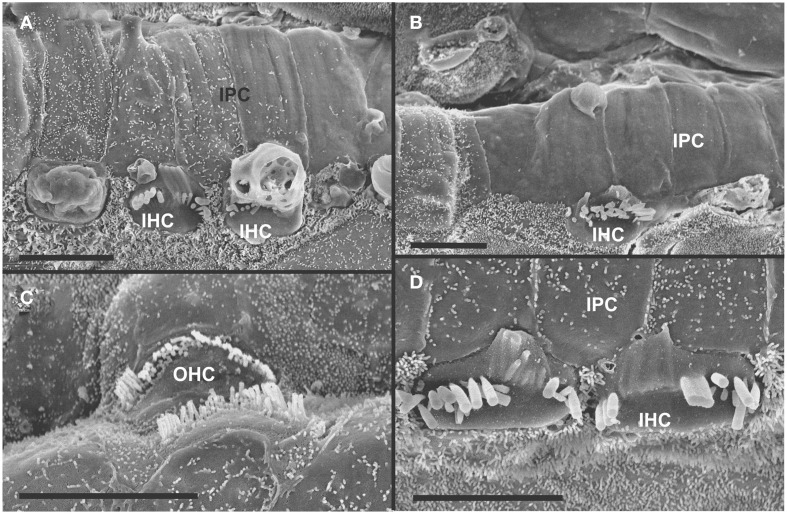
**Both IHCs (A,B,D) and OHCs (C) show variability in the length of stereocilia with partial or complete fusion and what appears to be resorption into the HCs**. Some IHCs show globular protrusions expanding into the scala media, occasionally from IHCs that bear some stereocilia **(A,B)**. Note that IPCs typically have very short microvilli **(A,D)** but, at places, may entirely lack microvilli formation **(B)**. Bar equals 5 μm.

**Figure 6:**
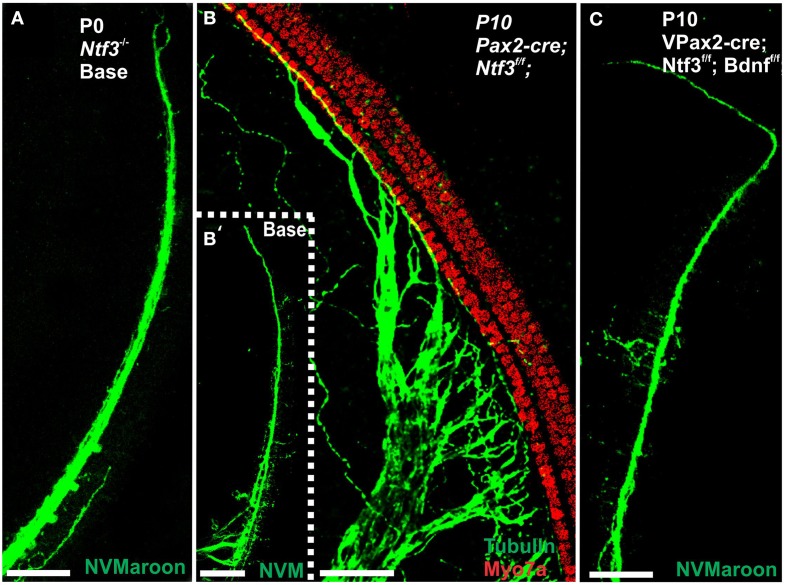
**Loss of innervation of the cochlear base is shown in mice with a complete deletion of *Ntf3* (A)**. A conditional deletion of *Ntf3* using *Pax2-cre* **(B)** or a conditional deletion of both *Bdnf* and *Ntf3* in the ear. Note that there is, at the most, a few fibers spiraling along the IHCs from the middle turn in either mutant and no matter the technique used (lipophilic dye tracing with NVMaroon, **A,C**; immunocytochemistry with anti-tubulin, **B′**). Importantly, conditional deletion of *Ntf3* using *Pax2-cr*e requires the additional elimination of one or two alleles of *Bdnf* before the phenotype approaches that of the unconditional null for *Ntf3* **(A,C)**. Note that the reduction of innervation has no apparent effect on early development of hair cells visualized with anti-Myo7a staining **(B)**. Bar equals 50 μm.

The organ of Corti of 4 month old mice with no innervation showed a nearly complete loss of almost all outer hair cell (OHCs) and over 60% of inner hair cells (IHCs) throughout the basal turn (Figures 3, 4). This contrasted with control littermates that showed a normal complement of three rows of OHCs and 1 row of IHCs with rarely any loss of HCs (Figure 3A, insert). The distribution of remaining HCs showed some aggregation near the very tip of the base but moving up only a few 100 μm toward the middle turn we found stretches of organ of Corti void of hair cells (Figures 3, 4). In fact, throughout most of the basal turn the OHC/supporting cell area had nearly disappeared in this mutant (Figures 3A–C) as compared to conrol littermates (Figure 3A, insert). Remaining OHCs showed reduced numbers of stereocilia with variable height, the more central stereocilia usually being much shorter compared to those more lateral (Figure 5C). The few remaining scattered inner hair cells showed a partial fusion of stereocilia, mostly organized as a single row with more or less extensive gaps between them (Figures 3, 4). Many remaining inner hair cells had only few stereocilia left on either side whereas other stereocilia showed various stages of shortening and fusion (Figures 4–6). Some IHCs showed a ballooning expansion protruding into the scala media, as previously described following otoxic treatment (Taylor et al., 2008), sometimes accompanied by barely recognizable stereocilia in the same cell (Figure 5A).

In some parts of the organ of Corti lacking any hair cells, the inner pillar cells also partially or completely dedifferentiated allowing continuity between the inner and outer spiral sulcus (ISS/OSS Figure 3B). We could not find a consistent relationship between loss of IHCs, OHCs and changes in inner pillar cells (IPCs). In some areas where all HCs were lost IPCs were near normal but were disrupted in others near remaining HCs (Figures 3B, 4C). The IPCs had the bundle of tubulin filaments protruding as little bumps due to the steep inclination of the IPC head toward the OSS. Where IHCs were lost, IPCs expanded laterally to fill the reticular lamina gap left by lost IHCs. These lateral expansions of IPCs either abutted the border cells (BC) of the ISS (Figure 3) or appeared to have a remaining layer of inner phalangeal cells with numerous short microvilli between the remaining IPCs and BCs (Figure 4). We presume these cells are remaining inner phalangeal cells (IPhC) as their numerous short microvilli resemble in detail those of IPhCs found between adjacent IHCs in areas that had IHCs. At places, the IPCs were partially dedifferentiated (Figures 3–5) leaving their heads standing freely over the remaining OPCs and the expanded OSS (Figures 3, 4). In other places we could not identify IPCs and BCs of the ISS seemed to approximate Claudius cells (CC) of the OSS (Figure 3B; asterisk).

Overall, the organ of Corti showed dramatic regional variation of cellular changes with profound variability along the length of the basal turn, ranging from stretches of nearly flat epithelium (Figure 3B) to near normal. Nevertheless, these changes imply a putative temporal progression toward the more differentiated middle turn: OHCs were lost more rapidly and the outer compartment dedifferentiates and was overgrown or replaced by CCs of the OSS. Many IHCs survived much longer compared to OHCs. Lost IHCs were replaced by expansions of the IPCs with or without retention of the inner phalangeal cells. IPCs were the longest remaining cells in the organ of Corti. When IPCs dedifferentiated this allowed BCs of the ISS to approximate CCs of the OSS, constituting what has been termed a flat epithelium (Izumikawa et al., 2008).

In summary, mutants lacking two neurotrophins allow the study of HC maintenance in the complete absence of any innervation (both afferent and efferent fibers) from approximately postnatal day 12 (P12) onward. Within the limits of the delayed loss of a few middle turn afferents and efferents, this model is consistent with some previous claims of HC development being independent of innervation. Consistent with the most controlled surgical approach to sever ear innervation (Favre and Sans, 1991), our data suggests that the HCs of the organ of Corti in mice have a survival capacity of around 100 days in the complete absence of any innervation from around P12 forward. Some OHCs and more near normal IHCs remain scattered between profoundly altered cellular organization of the OC indicates a large degree of local variation to the effect of postnatal loss of innervation. To further investigate the effect of limited innervation on long term HC viability, we next investigated hair cell viability using littermates with varying genotypes and long term maintenance of some innervation mainly to the middle turn of the cochlea.

### Complete absence of *Ntf3* and incomplete absence of *Bdnf (Pax2-cre; Ntf3^f/f^; Bdnf^f/+^)*

**Figure 7:**
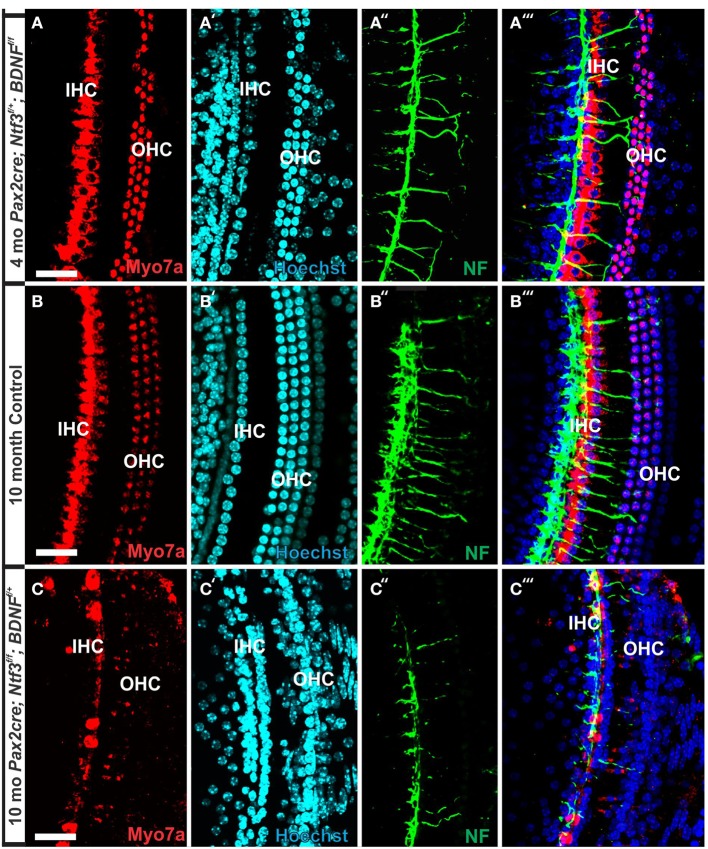
**Conditional deletion of *Bdnf* combined with heterozygosity of *Ntf3* using *Pax2-cre* leads to reduced innervation and sporadic OHC loss by 4 months (A″,A‴)**. In contrast, control littermates have a dense innervation and near continuous rows of IHC/OHCs, revealed with anti-neurofilament staining even at 10 months **(B″)** of the basal part of the middle turn. All remaining HCs stain positive for anti-Myo7a **(A–C)**. Note the regular appearance of HC nuclei with only an occasional OHC missing at this age **(B′)** compared to loss of nuclei in 4 months old partially denervated cochlea **(A′)**. The combined staining shows that nuclei (blue) and HCs (red) are the clear target of the many fibers **(B‴)**. In mutants lacking *Ntf3* and retaining only one allele of *Bdnf*, very few nerve fibers remain **(C″)**, many nuclei of OHC/IHCs are missing or are disorganized **(C′)**. Only few Myo7a positive IHCs or OHCs remain in mutants **(C)** and many have nearly undectable levels of Myo7a labeling **(C,C‴)**. Bar equals100 μm.

Previous work has shown that loss of a given neurotrophin has both a longitudinal and a radial effect. Loss of Ntf3 caused absence of basal turn spiral ganglion neurons with residual innervation spiraling along the inner hair cells from the middle turn spiral ganglion neurons (Figure 6). In contrast, loss of Bdnf caused only a reduced density of innervation in the apex with a reduction of afferents to the OHCs (Fritzsch et al., 2004; Yang et al., 2011). Either tubulin immunocytochemistry in neonates (Figure 6) or osmication in adults (Figure 2D) showed conditional deletion of Ntf3 with conditional deletion of only one allele of Bdnf resulted in loss of all spiral ganglion neurons in the basal turn. Middle turn spiral ganglion neurons had processes spiraling along the inner spiral bundle to the base. Even 10 month old mutants (Figure 7) had some fibers innervating mostly IHCs and mostly in the upper middle turn.

In contrast to control littermates, 10 month old *Pax2-cre; Ntf3^f/f^*; *Bdnf^f/+^* mutant mice showed no Myo7a positive staining throughout the basal turn (data not shown). We found Myo7a positive staining HCs in the middle turn and in the apex (Figure 7C). However, while control littermates (either no cre or no LoxP flanked neurotrophins) had near uniform Myo7a staining with occasional loss of one or two OHCs (Figure 7B), *Pax2-cre; Ntf3^f/f^*; *Bdnf^f/+^* mutants showed a profound reduction of Myo7a with very few, mostly IHCs normally labeled (Figure 7C). Hoechst nuclear staining confirmed the presence of OHCs and IHCs (and the occasional loss of OHC) in control animals (Figure 7B′) while nuclear stain was difficult to use to identify HCs in the mutant due to large gaps and distribution of nuclei at different levels (Figure 7C′). Numerous fibers could be traced to IHCs and OHCs in control animals (Figures 7B″,B‴) whereas very few tunnel-crossing fibers were found in mutants (Figure 7C″,C‴). These data suggest a progressive loss of HCs in the mutant. However, it needs to be stressed that areas exist in the middle turn of mutants with fairly normal HC distribution that seemingly correlated with apparent higher level of innervation density, though the details require more quantification. Since 10 months seemed to be on the advanced end of HC loss in these mutants, we concentrated the SEM study on the 8 month old mutants to learn more about the cellular changes to expand beyond the data obtained in double null neurotrophin mutant mice.

**Figure 8:**
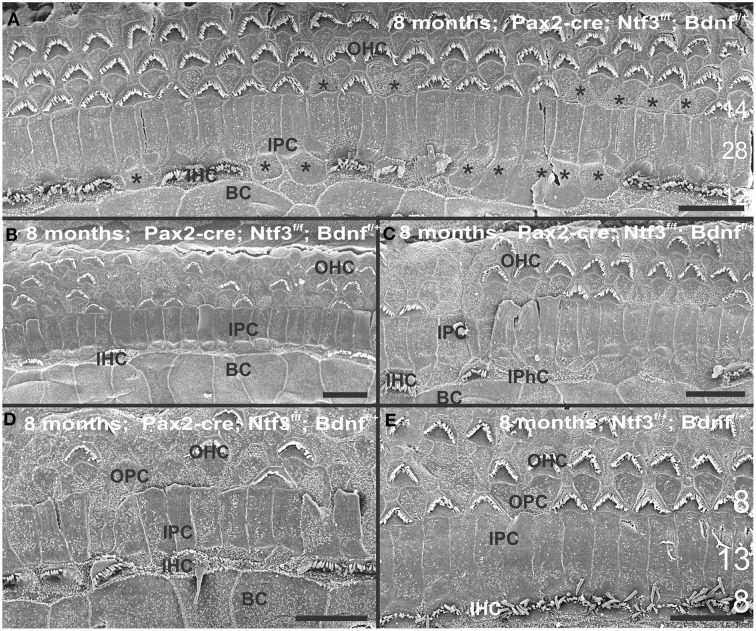
**The upper middle turn of an 8 month old *Pax2-cre; Ntf3^f/f^; Bdnf^f/+^* mutant mouse (A–D) is compared with a control littermate (E; no Pax2-cre)**. Mutant mice lack many OHCs and IHCs, sometimes as single cells and sometimes as partial rows (asterisks in **A**). In contrast, control animals show sporadic loss only of OHCs with a tendency to be more profound in the third row **(E)**. Spaces of lost IHCs are typically filled by medial expansions of IPCs that directly contact border cells (BC) of the inner spiral sulcus (ISS). Lost OHCs of the first row are mostly replaced by expansions of OPCs but sometimes by medial expansions of the first row of Dieter's cells. Due to the loss of OHCs and in particular IHCs, the ratio of IPC:OHC:IHC differs between control (13:8:8) and mutants (14:7:6). Bar indicates 10 μm

**Figure 9:**
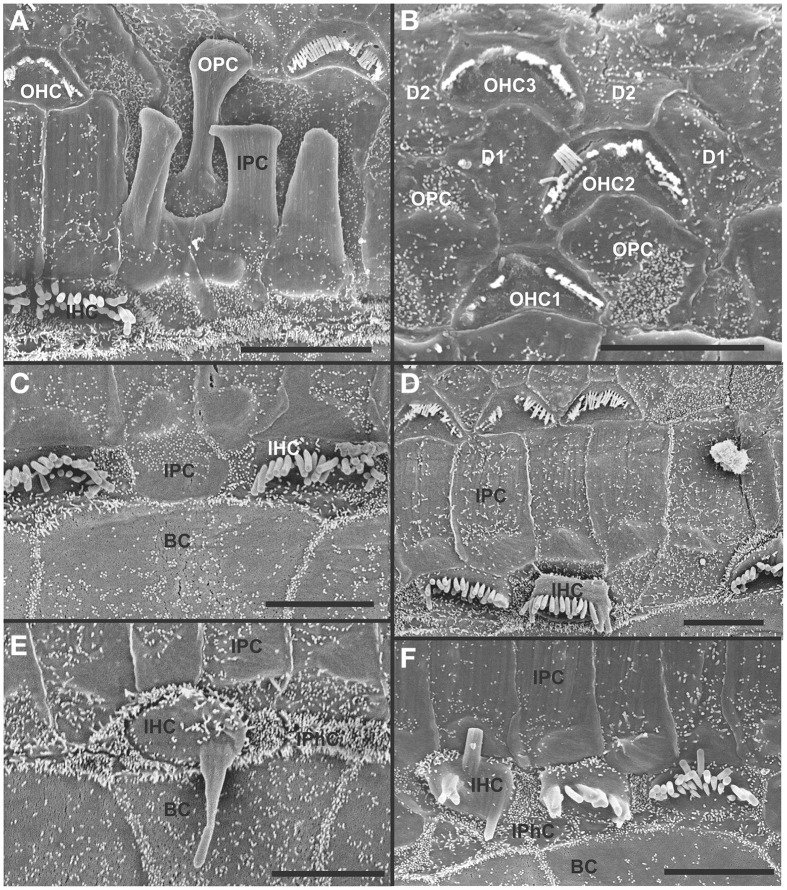
**Both OHCs (A,B,D) and IHCs (C–F) show various degrees of resorption and fusion of stereocilia in this 8 month old *Pax2-cre; Ntf3^f/f^; Bdnf^f/+^* as well as patchy loss and locally different degrees of reorganization of supporting cells**. IPCs always expand medially to close the reticular lamina over lost IHCs. However, IPCs may either directly contact **(C,D)** border cells (BC) or a layer of cells with numerous short microvilli, presumably inner phalangeal cells (IPhC), may be wedged between IPCs and BCs **(A,E,F)**. OPCs usually expand to complete the reticular lamina if the first row of OHCs is lost **(A)** but sometimes may expand to the second row of OHCs **(B)**. First row Deiter's cells (D1 in **B**) may occasionally expand to close the reticular lamina in places of lost first row of OHCs (OHC1 in **B**). Bar indicates 5 μm.

At 8 month, the SEM data revealed a less severe deficit compared to 4 month old, denervated cochlea (Figures 3, 8). Thus, a limited residual innervation maintains HCs under otherwise equal conditions for several more months compared to complete loss of innervation (Table 1). Most notable were differences in OHC vs. IHC loss and among the three rows of OHCs (Figure 8). Whereas in many cases there was no loss of OHCs in the third and second row, the first row of OHCs and in particular IHCs showed a regionally specific, severe loss (Figure 8A). This was in stark contrast to littermates that showed the well-known patchy loss of single OHCs that were scattered across all rows with a tendency to be more profound in the third row(Figure 8E). At places, most IHCs (Figures 8B,C) and nearly all OHCs of the first row (Figure 8D) were lost in mutants. In fact, in many instances, multiple IHCs were lost instead of the typical single OHCs (Figure 8). Thus, overall hair cell loss in the middle turn of mutants differed from age-matched littermates in showing loss of multiple adjacent HCs. Notably, there was virtually no loss of IHCs in control animals even at this age (Figure 8E). Closer examination showed fusion of multiple stereocilia in OHCs (Figure 9B) and IHCs (Figures 9C–F). This fusion in some IHCs was so advanced that only one or two prominent protrusions reached from IHCs into the scala media (Figures 9E,F). Loss of IHCs resulted in medial expansion of IPCs to either touch BCs (Figures 9C,D) or to leave inner phalangeal cells (IPhC) between them (Figure 9E). At places OPCs expanded into the outer compartment between the first rows of OHCs (Figure 9A). Loss of HCs in the first row of OHCs was usually filled by expansion of the lateral process of the OPCs (Figure 9A) but occasionally by a medial expansion of the first row of Deiter's cells (Figure 9B) generating a continuity of different types of supporting cells without any HC between them. This interpretation is consistent with a recent report showing that Deiter's cells function as scavengers that engulf dying hair cells (Anttonen et al., 2014).

**Table 1:** **Percent remaining hair cells quantified from three areas of 200 μm length near the base**.

**Genotype**	**% remaining HC 4 months (base)**		**% remaining HC 7–8 months (base)**	
	**IHC**	**OHC**	**IHC**	**OHC**
*Pax2-cre; Ntf3^f/f^; Bdnf^f/f^*	44%	8%	na	
*Pax2-cre; Ntf3^f/f^; Bdnf^f/+^*	na		51%	62%
*Pax2-cre; Ntf3^f/+^; Bdnf^f/f^*	na		11%	72%
*control; Ntf3^f/+^; Bdnf^f/+^*	100%	98%	100%	82%

*All data are means form 6 ears, except for the 4 months double null mutant that are from a single mutant*.

### Complete absence of *Bdnf* and incomplete absence of *Ntf3 (Pax2-cre; Ntf3^f/+^; Bdnf^f/f^)*

Previous work had demonstrated a limited effect of loss of Bdnf on cochlear innervation but complete loss of canal cristae (Fritzsch et al., 2004; Yang et al., 2011). Consistent with these embryonic data, we find no innervation left to the canal cristae of *Pax2-cre;* Ntf3^f/+^;*Bdnf^f/f^* (Figures 2E,E′). In contrast to the loss of vestibular innervation and a severe reduction of the vestibular ganglion (data not shown) the innervation of the organ of Corti was reduced (Figures 2F,F′). Interestingly enough, fewer fibers were present to the basal turn but without the profound loss of all basal turn afferents characteristic for mice null for *Ntf3* (Figure 2D). *Bdnf* expression changes from embryonic apical to neonatal basal expression (Flores-Otero et al., 2007) and more profound effects in the basal turn have been noted before in *Pax2-cre; Bdnf^f/f^* mice (Zuccotti et al., 2012). It appears that heterozygosity of *Ntf3* profoundly compounds the effect of simple loss of *Bdnf*, resulting in reduced innervation of the cochlea (Figures 2E,F). This confirms that long term loss of one neurotrophin combined with haploinsufficiency of the second neurotrophin increases innervation defects suggestive of previously proposed simple quantitative compounding effects (Yang et al., 2011). Each of these different combinations of partial Bdnf loss (*Pax2-cre; Ntf3^f/f^*; *Bdnf^f/+^*) or partial *Ntf3* loss (*Pax2-cre;* Ntf3^*f*/+^; *Bdnf^f/f^*) have clear differences in the remaining innervation (Figures 2D,F) pattern, despite the fact that in each case only one of the four neurotrophin alleles remains. We next confirmed the reduction of innervation in the *Pax2-cre; Ntf3^f/+^*; *Bdnf^f/f^* using immunocytochemistry (Figure 7A).

At 4 months the first effects of partial denervation in the basal turn appeared (Figure 7A). In fact, at this stage these mice already showed a loss of OHCs that was more obvious compared to a 10 month old control animal (Figures 7A,B). Specifically, multiple outer hair cells were missing, mostly in the base. As in other mutants, it appears that OHCs are preferentially missing in the first row compared to the second row (Figure 7). There was also some limited effect in IHCs which were less regular in their distribution, making them more difficult to assess by nuclear staining alone. Most of the remaining fibers that traced to OHCs showed features consistent with efferents (Figure 7A″). An occasional type II afferent fiber was identified (Simmons et al., 2011).

**Figure 10:**
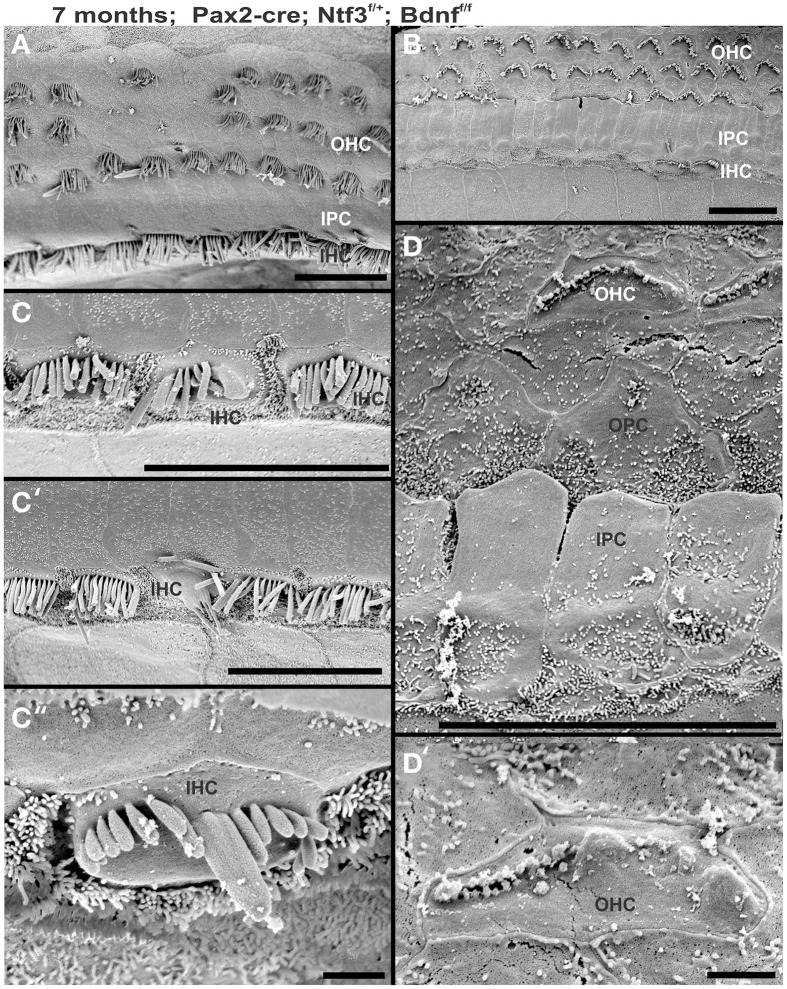
**Development of stereocilia and distribution of surviving hair cells is shown in a 7 month old *Pax2-cre; Ntf3^f/+^; Bdnf^f/f^* null mutant mouse**. Note that the apex shows a continuous row of IHCs **(A)** whereas the base has large stretches where there is only an occasional IHC left **(B)**. OHCs show loss in either region but the appears is more obvious in the innermost row of OHCs in the base whereas it is more obvious in the two outermost rows in the apex. IHCs show various unusual fusions of stereocilia **(C,C′,C″)** while others adjacent to these fused stereocilia appear normal. The few remaining OHCs in the upper middle turn show very short stereocilia **(D,D′)** if stereocilia are present at all. OHCs may show small bumps instead of stereocilia **(D′)**. Bar indicates 20 μm in **(A–C′)** and 5 μm in **(C″,D′)**.

Our SEM data mostly confirmed previous changes in mutants at a cellular level but also showed surprising longitudinal and radial effects. Most interesting was that stretches of IHCs were missing in the basal turn (Figure 10B) of 7 month old mice whereas all IHCs were usually present in the apex (Figure 10A). There were many losses of HCs in the second and third row of OHCs in the apex whereas the basal turn showed more losses in the first row. In particular, IHCs showed similar phenotypes in terms of fusion of stereocilia as previously encountered in the other mutations of this background (Figures 10C,C′,C″). Such fusion and reduced length of stereocilia was also found in OHCs were some cells showed short stereocilia on one side of the cell and only bumps of apparently fused stereocilia on the other side (Figure 10B). Overall, the changes in HCs and the pattern of loss with a large reduction in IHCs was similar to the other incompletely denervated mutant line analyzed here.

**Figure 11:**
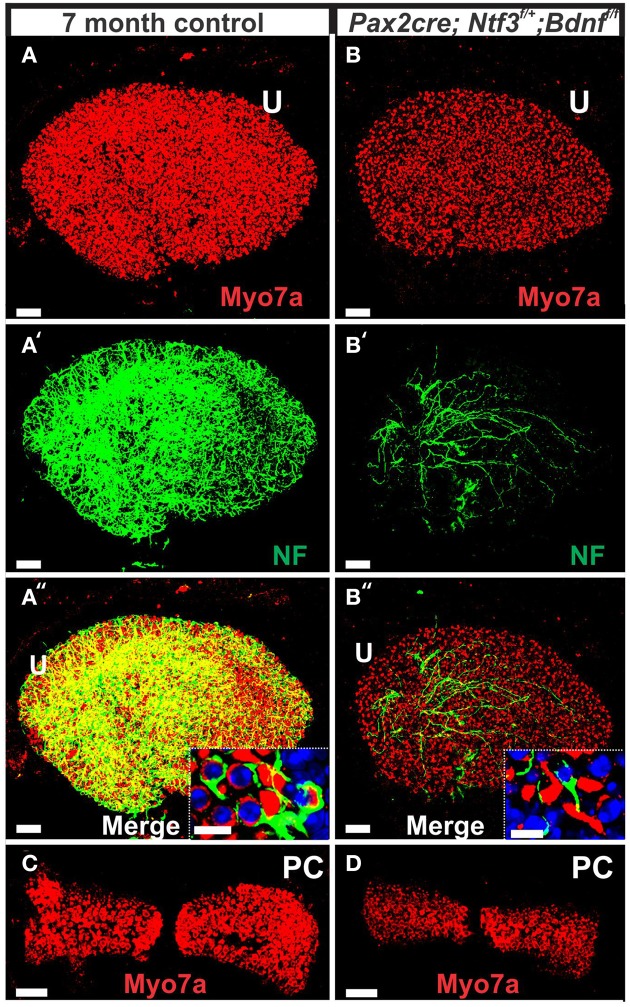
**This comparison of 7 months old control mouse vestibular organs (A,C) with a *Pax2-cre; Ntf3^f/+^; Bdnf^f/f^* littermate shows changes in size and innervation density**. Hair cells revealed with Myo7a **(A–D)** and their innervation **(A′,A″,B′,B″)** shows smaller sensory epithelia in mice lacking *Bdnf* and one allele of *Ntf3* (*Pax2-cre; Bdnf^f/f^; Ntf3^f/+^*) compared to the control littermate. Note that only the utricle (U) receives limited innervation in the mutant **(B′,B″)**. In contrast to the frequent calyces engulfing type I vestibular hair cells (insert in **A″**), mutants have only rare and partial calyces (insert in **B″**). The reduction in size of sensory epithelium is most profound in the posterior canal crista (PC in **C,D**) that is completely denervated and the only epithelium without any innervation throughout development. Bar indicates 50 μm **(A–D)** and 10 μm (inserts).

Mice without *Bdnf* have severe loss of all vestibular innervation at birth (Ernfors et al., 1995) but, in particular, the canal cristae loses all innervation (Fariñas et al., 2001; Fritzsch et al., 2004). Consistent with these known embryonic defects, there was no innervation of canal cristae at any postnatal stage (Figures 2C′,E′). Indeed, in 7 month old mutants we found only a limited innervation of the utricle but no innervation of the canal cristae (Figures 11A′,B′). There was also a noticeable reduction in size of the posterior canal cristae (Figures 11C,D) and the utricle (Figures 11A,B). Closer examination showed numerous calyces around type I vestibular hair cells in the control littermates but only a very rare calyx in the mutants (inserts in Figures 11A″,B″) consistent with a previous report that calyx formation requires normal Bdnf signaling through the TrkB receptor (Sciarretta et al., 2010).

**Figure 12:**
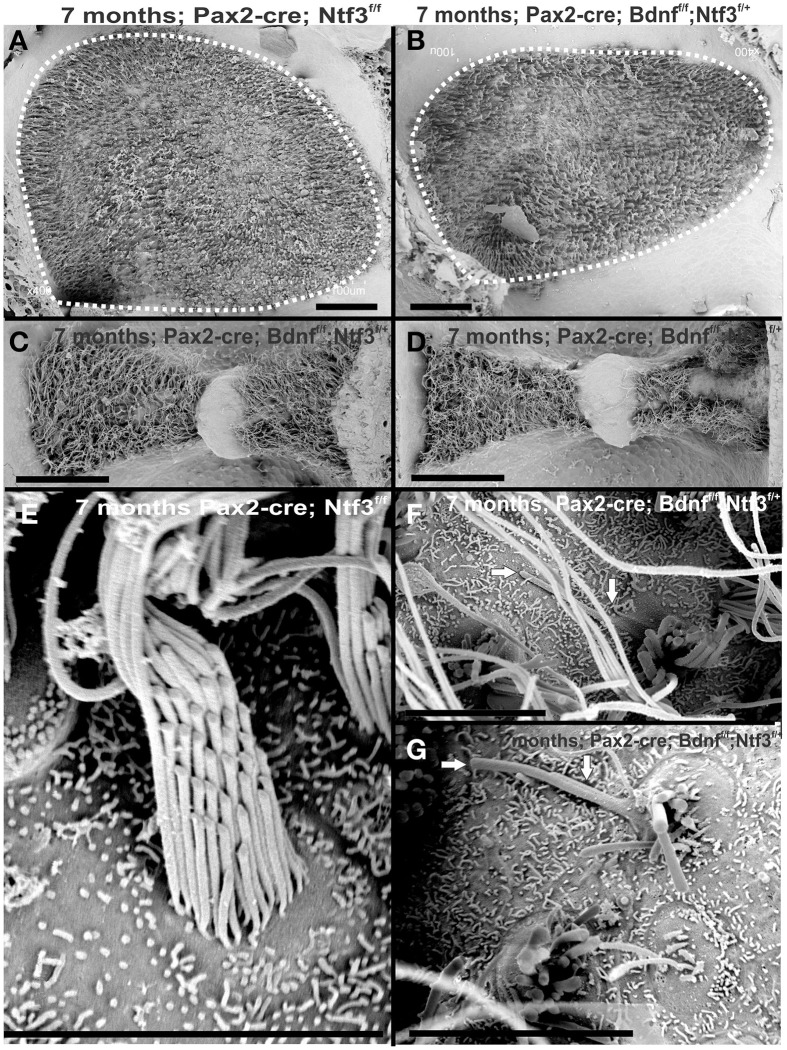
**The 7 month old mice lacking *Bdnf*and one allele of *Ntf3* (*Pax2-cre; Bdnf^f/f^; Ntf3^f/+^*) by *Pax2-cre* shows reduced size of sensory epithelia most obvious in a comparison of the utricle with that of a control littermate (dotted line in A,B)**. Interestingly, there is a size difference in the mutant between the anterior canal crista **(C)** and the posterior canal crista **(D)**. While the anterior canal crista is close to the remaining innervation of the utricle in this model and may transiently receive limited innervation, the posterior canal crista is removed from the limited innervation in the basal turn of the cochlea. Vestibular hair cells in the control animal show the normally bundled organization **(E)** whereas many aberrant bundles are found in the hair cells of the posterior canal crista around the “balding” region shown in the right hemicrista **(D,F,G)**. These hair cells show splayed bundles of stereocilia with fused stereocilia, and stereocilia of variable length that occasionally appear to be lying flat on the remaining epithelium (arrows in **F,G**). Bar indicates 50 μm **(A–D)** and 20 μm **(E–G)**.

SEM data also suggested a smaller utricular area compared to the control littermates. Only minor changes were found in HCs such as incomplete stereociliary bundles. However, such changes were difficult to document due to the density of stereocilia in the utricle. However, the posterior canal cristae appeared reduced in size compared to the anterior canal crista (Figures 11C,D) and had stretches of hair cells without long stereocilia (Figure 12D), consistent with gaps in HCs shown by immunostaining (Figure 11D). Some of the HCs in these areas had partially fused stereocilia (Figures 12F,G) that were lying flat on the surface of the epithelium (arrows in Figures 12F,G). Bundles were composed of stereocilia of uneven size and uneven length. Further quantification is needed to verify how much of the obvious shrinkage is due to loss of calyces and/or due to hair cell loss.

**Figure 13:**
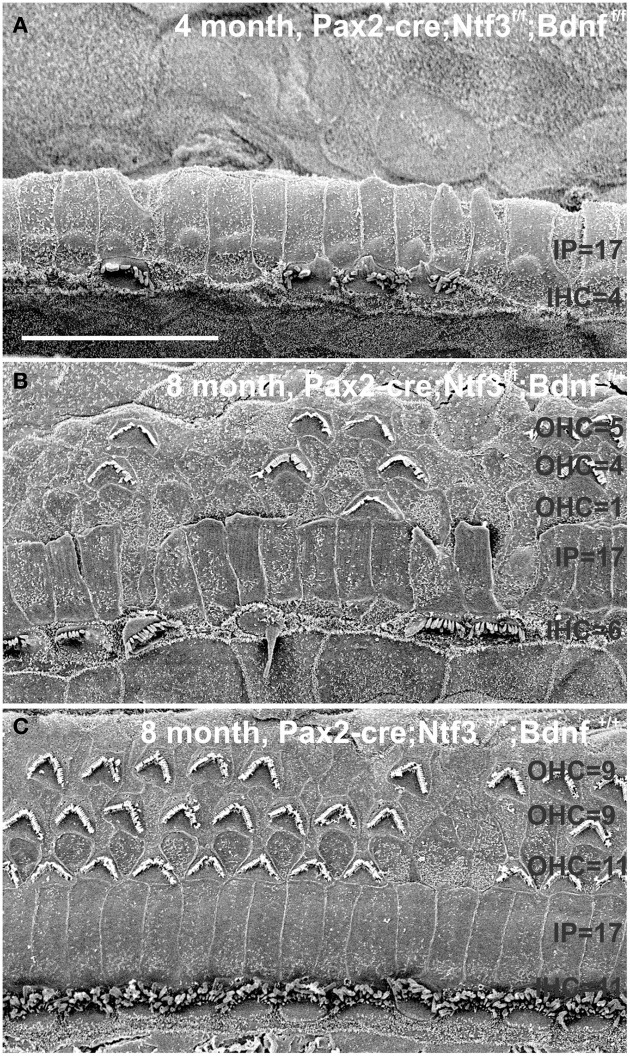
**This comparison shows the pertinent differences in complete loss of the entire outer compartment and nearly all OHCs in a 4 month old double neurotrophin conditional null mouse (A) as compared to a 8 month old mutant that retains one allele of *Bdnf* (B) and a 8 month old littermate control (C)**. Note that all mice have about 17 IPCs but a variable number of HCs. Control littermates retain all IHCs for at least 8 months, forming an approximate 4:3 ratio of IPCs and IHCs **(C)**. This changes to a 4:1 ratio in double null mutants **(A)** and a 3:1 ratio in partially denervated mice **(B)**. Note the variable loss of OHCs that is most profound in the first row in a mutant with incomplete loss of innervation **(B)** whereas it is more profound in the second and third row in control littermates. Bar equals 20 μm.

In summary, changes in HCs after partial denervation require at least twice as long to develop compared to complete denervation (Figure 13; Table 1). The overall changes at the hair cell level are somewhat similar and consist of fusion of stereocilia and shortening, both in IHCs and OHCs (Figure 13) and the vestibular epithelia (Figure 12). The reorganizations of the remaining supporting cells is more obvious in the organ of Corti and shows medial expansion of IPCs into the territory of lost IHCs and lateral expansion of OPCs into the territory of the lost first row of OHCs. The simple fact that in our mixed background we find profound loss of IHCs even with partial denervation, combined with the unusual phenotypes of reduced Myo7a immunopositivity, and fusion of stereocilia suggests that these effects are mediated by yet to be determined compounds associated with innervation.

## Discussion

### Denervation defects HCs

Overall, our data suggest a time line of innervation dependency of cochlear HCs of ~4–8 months with loss of all OHCs and many IHCs of the basal turn in the absence of any innervation at 4 months (Figures 3, 4, 13). This is within the same range previously reported for the vestibular HCs after transection of the vestibular nerve without compromising the blood supply (Favre and Sans, 1991). In contrast, in mice, most vestibular HCs require at least 7 months of complete denervation before noticeable changes can be identified (Figure 12). The time line of several months of viability of denervated hair cells also agrees with published data on the lateral line mechanosensory cells in salamanders and frogs (Jones and Singer, 1969). Different to these obvious effects on long term maintenance, both *in vitro* and *in vivo* data clearly demonstrate that maturation and short-term survival of inner ear HCs is possible in the complete absence of any innervation (Fritzsch et al., 1998, 2005b). Our data confirm a normal complement of HCs at P12 even when little innervation remains (Figures 1, 7). Contradictory data should be reconsidered in the light of partial and/or difficult to detect remaining innervation and the time lapse between denervation and analysis as well as the time at which denervation is initiated (Sugawara et al., 2005). Loss of hair cells in complete denervation cases should not be dismissed as likely due to blood supply problems (Sugawara et al., 2005; Suzukawa et al., 2005) as blood supply is not an issue in our mutant mice.

Recent data suggest that altered synaptic activity can induce inner ear HC loss over a long period of time (Kidd and Bao, 2012) but does not show a clear overall correlation between loss of HCs and loss of neurons (Perez and Bao, 2011). Most recently physiological defects were found in OHCs after long term efferent disruption (Liberman et al., 2014). The molecular basis of neurotrophic support from sensory epithelia to sensory neurons is well-known (Fritzsch et al., 2004; Bailey and Green, 2014). Neither the molecular basis of afferent support on developing auditory nucleus neurons (Levi-Montalcini, 1949; Rubel and Fritzsch, 2002) nor the molecular basis of innervation on the physiology of HCs (Liberman et al., 2014) or the long term viability of hair cells (Figure 13; Table 1) is known. The fact that neurons die after embryonic (Pan et al., 2011) or adult HC loss in rodents (Alam et al., 2007) but not in humans (Linthicum and Fayad, 2009) indicates some yet to be molecular defined species-specific differences. Adding to this emerging complexity of adult HC-SGN interactions are recent data on loss of afferent innervation and SGNs after frequent sound exposures that seemingly does not affect HCs (Kujawa and Liberman, 2009), at least not if the neuronal loss spares over 10% of the SGNs (Makary et al., 2011). Evaluating our model in other mammalian species could verify if the effects described here are unique to the genetic background of our conditional deletion mice or can be expanded to other mammals or even humans. Previous work on dependency of cochlear nucleus neurons on innervation shows a profound critical phase and delayed loss of innervation has progressively less effects on cochlear nucleus neuron viability (Rubel and Fritzsch, 2002). Our denervation experiment is certainly earlier and more complete compared to other attempts and our effects could indicate a critical phase of hair cell dependency on innervation. The longer viability of hair cells in partially denervated mice could indicate that targeted deletions of neurotrophins at different time points are needed to exclude other interpretations. Available evidence suggests presence of neurotrophin receptors only on neurons (Ylikoski et al., 1993; Fariñas et al., 2001) but delayed expression of limited receptors needs to be verified using appropriate modern techniques to rule out any possible direct effect. Different cre lines such as a combination of Atoh1-cre (Matei et al., 2005) with induced delayed deletion in supporting cells using Fgfr3-creER (Anttonen et al., 2014) could result in more viable mice lacking all inner ear innervation. Another way to achieve denervation without affecting blood supply is by chemical treatment such as ouabain (Yuan et al., 2014). However, in this model type II neurons and efferents remain and HC are sensitive to ouabain (Fu et al., 2012).

### Limited innervation can provide long term HC support

Our data and those gathered in other systems (Fritzsch et al., 1998) raise the possibility that compromised neuronal viability provides some feedback for long-term integrity of mechanosensory HCs in the inner ear but apparently with a large time delay, that is even longer with a limited innervation of less than 10%. We base this suggestion on quantification of spiral ganglion neuron loss in *Ntf3* null mice (~85%) and *Bdnf* null mice (~7%) that combines to ~92% loss of SGNs (Bianchi et al., 1996; Fariñas et al., 2001). Assuming that there is a simple additive effect, this suggests that most papers claiming no effect of severe reduction of innervation on hair cell viability need to be revisited to determine exactly how much innervation was left when HCs appear to be normal and at which age all innervation was indeed lost. In addition, as innervation falls below 10% it appears that a very profound time delay exists before HCs are compromised that would be problematic for many studies dealing with mice that show premature age related HC loss. How this support of HCs is distributed between efferents and afferents remains to be elucidated but data on other sensory systems without efferents clearly point out the importance of afferents (Fritzsch et al., 1990). In fact, the unusual feature of our model is the effect on IHCs which receive only transient innervation during development and in certain circumstances in the adult system (Simmons et al., 2011; Lauer et al., 2012). Therefore, for IHCs, it appears likely that their high density of afferent innvervation plays a major role (Fritzsch et al., 2015). Such an interpretation is consistent with the preferential IHC loss in our models with diminished innervation. The apparent preferential loss of the first row of outer hair cells could relate to the difference in innervation density between IHCs and OHCs, assuming that afferents release a diffusible factor that can reach a short distance comparable to neurotrophins (Fritzsch et al., 2004).

### Reconciling literature discrepancies

Cutting the cochlear nerve has led to contradictory, variable and inconclusive data (Sugawara et al., 2005) possibly due to a difficult mix of surgery related blood supply disturbance and incomplete elimination of all innervation, differences between experimental animals and the possible effect of a critical phase of HCs on innervation. We reason that all these data could be reconciled if it could be established that mechanosensory HCs of the ear depend on a yet to be defined critical threshold of afferent and efferent innervation during a critical phase, comparable to other sensory cells (Fritzsch et al., 1998) and cochlear nucleus neurons (Rubel and Fritzsch, 2002). However, neuronal dependency may take a longer time to manifest itself in the case of mechanosensory HCs of the ear compared to other sensory systems (Favre and Sans, 1991) but is comparable in its timeline to the mechanosensory lateral line system (Jones and Singer, 1969). Consistent with our anatomical data, long-term viability (Walsh et al., 1998) and function of outer hair cells (OHCs) might depend on efferent innervation (Liberman et al., 2014), whereas minor alterations in synaptic transmission may affect viability of inner hair cells (IHCs) exposed to loud sound (Zuccotti et al., 2012). Ideally, one would like to eliminate a neurotrophic factor (Lindholm and Saarma, 2010; Bailey and Green, 2014) to show effects we describe here in the presence of innervation, comparable to the loss of spiral ganglion cells in neurotrophin mutants in the presence of hair cells (Fritzsch et al., 2004) to verify the nature of this molecule (or molecules). Whether such molecules could also be the basis for the unknown support of developing cochlear nucleus neurons (Levi-Montalcini, 1949; Rubel and Fritzsch, 2002) remains to be seen.

### Partial denervation may aid in the study of age-related HC loss

This mouse model, with no or with greatly diminished innervation, allows us to test the hypothesis that cochlear HCs depend on innervation but have a different time constant compared to other sensory systems and that vestibular HCs are even more resilient. Unfortunately, our partially denervated model is more difficult to interpret. There is a well-known dependency of cochlear innervation on support provided by the normally developed organ of Corti (Bailey and Green, 2014). This support will obviously decline as the organ of Corti dedifferentiates upon loss of HCs (Alam et al., 2007; Pan et al., 2011, 2012). It is possible that additional loss of innervation due to loss of HCs may accelerate regionally specific HC loss (a possible negative feedback loop). However, this is of no concern for the general problem investigated here, namely that mechanosensory HCs depend on a limited level of innervation for long-term viability. Such feedback loops have not been apparent in previous work simply because the loss of neurons in most cases studied over long periods is far less (Makary et al., 2011) compared to our presumed 93–100% loss of neurons. Only sparing HC loss has been reported after efferent deletion (Walsh et al., 1998), in contrast to our massive loss of HCs within 4 months after all afferents and efferents have been deleted (Figure 13). More recent work has suggested the existence of such a feedback loop regulating functionality of OHCs after complete elimination of efferents (Liberman et al., 2014). Unfortunately efferents depend on afferents for homing (Simmons et al., 2011) and thus early afferent loss will result in efferent loss as well. Our model thus cannot easily distinguish between afferent and efferent long term support.

### Comparison to models of induced HC loss

Overall, the loss of hair cells and reorganizations of the organ of Corti to eventually turn into a flat epithelium presented here follows, to a large extent, changes observed after aminoglycoside treatment (Taylor et al., 2012, 2008). However, while hair cells are lost rapidly after aminoglycoside treatment (Taylor et al., 2008) or carboplatin treatment (Ding et al., 2012) it takes weeks to months (depending on the mouse line) for the organ of Corti to reorganize (Taylor et al., 2012). In the case of complete denervation, we see progression of HC loss and reorganization over several months with profound local variation. Our data suggest that there is a correlation with changes in Myo7a expression (Figure 7) and fusion or resorption of stereocilia (Figure 9) that resembles the degeneration of HCs in Myo7a mutant mice (Self et al., 1998). This similarity is even more profound in Myo6 mutants that show fusion of stereocilia of IHCs and OHCs (Self et al., 1999; Hertzano et al., 2008) remarkably similar to our data (Figures 5, 9, 10, 12). Conditional deletion of Cdc42 leads preferentially to IHC loss through stereocilia fusion (Ueyama et al., 2014) that is nearly identical to mice with incomplete denervation. However, while Cdc42 mice lose IHCs after 8 weeks, IHCs take over 4 months to degenerate even in completely denervated mice. It remains unclear if this correlation indicates some causality. If so, it could indicate that altered expression of Myo6, Cdc42 and other proteins associated with stereocilia homeostasis (Kitajiri et al., 2010; Sekerková et al., 2011) could underlie not only innervation dependent hair cell maintenance but may play a role in age dependent, variable loss of HCs through interference with the stereocilia homeostasis (Lenz et al., 2010). Like age dependent HC loss (Otte et al., 1978; Keithley and Feldman, 1982; Wright et al., 1986), loss of HCs in our model is highly variable with rather different time lines for individual HCs that is even more different with residual presence of some innervation. Combined, our data strongly support the conclusion of local variability of effects after aminoglycoside treatment (Taylor et al., 2012) and support the argument raised by Taylor et al. (2012) that attempts to regenerate an organ of Corti requires a close look at this variability of local effects. Given the variable residual presence of IPCs, IPhCs or nothing, strategies must be designed to take the local variations in supporting cell viability into account when attempting to restore a functional organ of Corti. Our data support the notion of molecular uniqueness and independence of IPCs (Fritzsch et al., 2015) that have been shown to survive long term in other molecular models of HC loss (Pauley et al., 2008) as well as in our model (Figure 13).

### Translational aspects

Previous work has shown that subcritical sound levels can result in long-term loss of some innervation (Kujawa and Liberman, 2009). It is possible that such retraction of nerve fibers is accelerated through compromised neurotrophin signaling (Wang and Green, 2011), accelerating additional loss of innervation caused by sound. Such a possible loop can eventually result in loss of HCs as consequences of mutation related to reduced signaling of neurotrophins or other molecules such as Igf-1 (Varela-Nieto et al., 2013) combined with reduced density of innervation. Given our data and those after aminoglycoside treatment that indicate profound local variations (Taylor et al., 2012), any treatment of the aging human cochlea to retain HCs or restore the organ of Corti needs to take local variation into account. This model could be used to develop expression profiles of remaining HCs to eventually identify genes (Liu et al., 2014) responsible for their viability focusing on interactions between Cdc42 and Myo6, the two mutants (Self et al., 1999; Ueyama et al., 2014) that have the greatest similarities with our denervation hair cell phenotype. Ultimately, we would need to identify the molecular basis of the neuronal signal to generate small molecular analogs to rescue HCs in the absence of innervation. Several candidate trophic factors exist (Bailey and Green, 2014), some with unknown function or expression in the ear such as MANF (Lindholm and Saarma, 2010). Such molecules could possibly enhance viability of innervated HCs, rescuing them for longer periods to provide the growing aging population of the world with functional HCs for hearing and balance.

### Conflict of interest statement

The authors declare that the research was conducted in the absence of any commercial or financial relationships that could be construed as a potential conflict of interest.
